# Injury Prevention: Freestylers’ Awareness of FIS Code of Conduct for Snow Parks

**DOI:** 10.3390/ijerph17010308

**Published:** 2020-01-01

**Authors:** Luis Carus, Isabel Castillo

**Affiliations:** 1 Faculty of Health and Sport Sciences, University of Zaragoza, 22002 Huesca, Spain; 2 Faculty of Business and Public Management, University of Zaragoza, 22002 Huesca, Spain; icastillo@unizar.es

**Keywords:** injury prevention, freestyle, snow park, FIS Code of Conduct for Snow Parks

## Abstract

The objectives of the present study were to assess general perceptions of safety in snow parks (SPs), general knowledge of rules existence, and both active and passive awareness of the International Ski Federation (FIS) rules contained in its Code of Conduct for SPs in order to define target groups for injury prevention-specific education interventions. Data were drawn from 436 freestylers randomly interviewed. The study was conducted during the 2018–2019 winter season in the SP of a major winter resort located in the Spanish Pyrenees. A questionnaire assessing personal data (gender, age, gear used, self-reported skill, and frequency of use), general perceptions on safety, general request for rules, and awareness of existing rules in SPs was developed. Chi-square goodness-of-fit tests were used to compare characteristics between groups. It was revealed, for accident prevention purposes, a concerning general lack of knowledge of existing rules in SPs (63% of participants ignored them). Risk-inducing situations that could result in severe injuries, such as familiarity with the right progression in choosing features and/or stunts or with safety equipment, were largely assessed incorrectly (94% and 70% of participants, respectively). Appropriate intuitive behavior increases with experience: youths and beginners are less able to implement FIS rules than more experienced freestylers.

## 1. Introduction

Primary prevention, including the identification of risk factors and the promotion of safety behavior, is the first step towards systematic injury prevention in sports [1]. Injury prevention involves analyzing the magnitude of the problem and identifying behavioral risk factors [2]. Past research has reported that a lack of knowledge of safety rules prevails among injured skiers rather than among non-injured [3]. 

Alpine skiing and snowboarding on traditional slopes are associated with a risk of injury and skiers’ and snowboarders’ risk of injury is dependent on their knowledge of proper behavior on the slopes [3,4,5]. Therefore, regulation of skiing and snowboarding slope activities is essential to have users informed in order to reduce accident and injury rates [6]. Few studies have evaluated the effectiveness of skiing and snowboarding instruction in increasing safer behaviors and reducing the risk of injury, and only one has assessed knowledge of existing rules for regular slopes [6,7,8,9].

As snowboarding became more prevalent on ski slopes, ski resorts began to develop man-made features, mainly quarter and half pipes, specifically to attract snowboarders, though skiers soon joined them in the new features to practice what was to be known as freestyling [10]. Their success led resorts to define specific areas, now known as Terrain or Snow Parks (SPs), in an attempt to address safety issues by avoiding accidents due to collisions between these new freestylers and regular slope users [10]. SPs are specific playgrounds containing different types of aerial (“jumps”) and non-aerial (“jibs”) man-made features that allow freestylers to perform a wide variety of maneuvers and stunts known as freestyling [11,12]. 

Before long, resorts were competing to create the best SPs, and the vast majority of ski resorts around the world now have one [10,11]. However, freestyling in SPs involves serious safety risks to ski and snowboard freestylers [10]. Previous studies have suggested an association between SPs and an increased risk of injuries, and specific ones comparing skiing and snowboarding injuries sustained in SPs with those sustained on regular slopes provide evidence that SP activities increase the risk of severe injury compared to on-slope activities [11,13,14,15,16,17]. Therefore, it is imperative to inform freestylers of the rules and regulations of SP activities in order to contain and reduce accident and injury rates [18].

With the aim of promoting safe skiing on traditional slopes, in as early as 1967 the International Ski Federation introduced rules that applied to skiers [19], and later on, with the consolidation of snowboarding as an alpine discipline, updated them in order to embrace the enthusiasts of the new sport [20]. However, it wasn’t until September 2018 that, to promote safe freestyling in SPs, the FIS, in its Code of Conduct for Snow Parks [21], introduced rules that specifically apply to all freestylers (Table 1). Each freestyler, either skier or snowboarder, is expected to be familiar with these rules prior to freestyling [21]. In the event of an accident resulting from non-compliance with any of the FIS rules, it is possible that a claim for damages may be made, and all skiers and snowboarders, whether freestylers or not, should be aware that these rules are given significant weight in legal proceedings [22].

To strengthen intervention programs for safety, it is essential to include behavior modification. Inappropriate and hazardous behavior may be intentional or a consequence of a lack of knowledge [6]. 

Even though it is common practice among Spanish ski resorts to display local rules at the entrance of their SPs, at present the complete set of rules contained in the 2018 FIS Code of Conduct for Snow Parks can neither be found on other mediums, such as lift tickets, annual pass waivers, resorts maps, or on boards within or in the surroundings of the SPs, nor on the websites of key institutions such as the Snow Sports Spanish Federation or the Spanish Association of Ski resorts, which makes the FIS website the only conspicuous way for SP users to gain access to the Code of Conduct for Snow Parks.

The aim of the study was to investigate freestylers’ general perceptions of safety in an SP, general knowledge of rules existence, and both active and passive—understood as intuitive behavior in a given situation in the SP—actual knowledge of FIS regulations. These data, in correlation with freestylers’ gender, age, self-reported skill level, gear used, and frequency of use of the SP, may contribute to defining the target groups for specific education interventions. 

## 2. Methods 

This study was conducted following the approval of the University of Zaragoza research board. The research was carried out following the rules of the Declaration of Helsinki regarding research involving human subjects (revised in 2013 in Fortaleza, Brazil). The data were gathered through a survey performed between December 2018 and April 2019 in different weather conditions and at different times of the day, at the SP of a major resort in the Spanish Pyrenees with information provided by a random sample of users aged 12 (SPs access minimum age) or over. 

The SP was separated from the other ski runs, and its layout and the type and number of features did not vary during the season. It consisted of eight aerial features (1 half-pipe, 1 big jump [~4 m], and 6 jumps [∼1 m]), and the same number of non-aerial features (2 flat rails, 1 c-rail, 4 flat boxes, and 1 rainbow box). The number of features, as well as their characteristics, such as height, width, or length, overall coincided with those found at renowned SPs across the mountain range, such as the ones managed by ski resorts located in the Andorran Pyrenees. At the entrance of the SP the resort assigned a difficulty rating to individual features, but made no reference to rules or recommendations. 

The survey was conducted by experienced interviewers who had received specific instruction for the task on matters such as where to approach interviewees (e.g., entrance or exit), how to make them comfortable (e.g., their faces to the sun or backs to the wind), or how to conduct the interview safely (e.g., places not to be stopped at).

Similar to a questionnaire previously devised and validated to collect data on knowledge of existing rules among slope users [6], ours was designed to assess personal data—gender, age, gear used (“skis/snowboard”), self-reported skill level (“beginner/intermediate/expert”), and frequency of use of the SP (“occasionally/frequently/always”)—, general perceptions of safety (“How safe do you feel in the SP?”), perceived importance of rules (“Are rules important in the SP?”), general knowledge of existing rules (“Do specific rules for the SPs exist?”/”Can you provide the name for these rules?”), and actual, active, and passive—intuitive behavior for given situations in the SPs (Table 2)—knowledge of the FIS rules for SPs. 

Except for the open question regarding actual active knowledge of existing rules devised to assess those the participants knew (“Can you please mention the rules you know?”), questions were closed, with “very safe/safe/unsafe/very unsafe” as possible choices to assess responses on perceptions of safety; “important/unimportant” as possible choices to assess responses on perceived importance of rules; and correct (“yes”) or incorrect (“no” or “I don’t know”) as possible choices to assess responses on questions related to general knowledge of FIS Rules and those related to passive knowledge. Only those participants who acknowledged knowing the existence of general rules for SPs were asked about their ability to provide the proper name of these rules and to mention the existing rules they knew.

The design of the survey followed best practices as described in Vaske [23] and Dillman et al. [24]. Setting personal data aside, in order to ensure construct validity and to reach a compromised solution between the inclusion of determinant items and a practical length for the questionnaire to be used in the empirical research, we obtained feedback from 15 experts (1 SP manager, 3 SP patrollers, 1 ski patrol officer, 1 ski patrol practitioner, 1 former coach of the Spanish national freestyle team, 4 Level III snowboard certified instructors, and 4 Level III alpine skiing certified instructors) to define the final list of questions [23]. When the experts who constituted the panel were first contacted, they were asked to submit an account of the wording of every item that they thought, according to the best of their knowledge and professional experience, could assess users’ general perceptions of safety and knowledge of the FIS rules for SPs. After two consultation rounds, a consensus was reached on the questions that were finally included. 

Tables were used to display both items, expressed as total values and percentages or percentages alone, to provide descriptive analysis, and results of Pearson’s chi-squared tests were used to assess independence between groups within gender, age, gear used, skill level, and frequency of use of the SP. It was considered statistically significant a *p* < 0.05 level. Data were analyzed using Minitab 19 (State College, Pennsylvania, USA) for Windows. 

## 3. Results

Table 3 presents a prevailing profile corresponding to the following characteristics: more than half of the respondents were male (68%), between 25 and 49 years of age (61%), freestyle snowboarders (57%), self-reported intermediate skill level (51%), and frequent users of SPs (58%).

As shown in Table 4, freestylers reported feeling safe (48%) or very safe (32%) while jumping and jibbing in the SP (Table 4). A limited number (17%) of them acknowledged feeling unsafe, while only a few (3%) felt very unsafe. Specifically, seniors felt less safe than adults and youths, and the latter felt particularly safe (*p* < 0.001). Those who use the SP occasionally (46%) felt unsafe while those who use it always (49%) felt very safe (*p* < 0.001). Gender, gear used, and skill level of the participants did not have a significant effect on their sense of safety.

As many as 72% of respondents thought rules in the SP were important (Table 5). Both men (67%) and women (83%) predominantly agreed with the statement that rules are necessary in SPs. There was a significant difference in the three age groups’ responses to this statement: adults (85%) and seniors (97%) were more likely to agree that rules are necessary, whereas youths (60%) were more likely to disagree (*p* < 0.001). The difference in the three ski level groups was also significant (*p* < 0.001), being that the experts were the most likely group to perceive rules in SPs as important. Referring to gear used and frequency of use of the SP, the results showed no significant differences.

Differences regarding the knowledge of general rules existence and the ability of freestylers to provide their correct name are shown in Table 6. Well under forty percent of respondents (37%) were aware that general rules for SPs exist, and 16% of them (6% of the sample) could provide the proper name for these rules. Age had a significant bearing over the knowledge of the existence of general rules for SPs. Only 28% of the youngest participants knew about the existence of rules for SPs against 40% of adults and 44% of seniors. It is noticeable that in none of the three groups participants reached 50% of correct answers. Gear used, skill level, and frequency of use also had a significant influence on the knowledge of the existence of general rules for SPs. Remarkably, beginners revealed a large deficit in knowledge, whereas skiers and those who always use the SP displayed the most knowledge in their respective groups. The term “FIS rules” was more familiar to seniors and to freestylers with the best freestyling ability, while beginners, intermediates, and those who use the SP only occasionally were able to identify it.

Among those who knew that rules for general application in SPs exist, regardless of whether they acknowledged their name (FIS Rules) or not, the most common responses (Table 7) were the third (“Terrain inspection”) and the ninth (“Assistance”) rules. Between forty and fifty percent of respondents mentioned the fourth, seventh, and eighth rules. The second (“Training with others”), sixth (“Progression”), and tenth (“Identification”) rules were rarely identified. 

The influence of age proved significant in reference to the mentioning of some rules, specifically, rules one, three, five, eight, and nine, were more accurately identified by seniors over adults and youths. The influence of skill level also proved to be significant, with experts being much more accurate than beginners and intermediates when acknowledging rules seven, eight, and nine. Gear used had a significant influence when participants quoted rules one, three, four, and seven, with skiers showing more knowledge over snowboarders (rules one and four) or vice versa (rules three and seven). Similarly, with respect to frequency of use, significant differences were found between groups, with some groups showing better knowledge over the others: rules three and four were better identified by occasional users, rule seven by frequent users, and rule nine by those who always use the SP. Gender had no significant influence, except for rule four, which was better acknowledged by males than females.

Differences regarding freestylers’ passive knowledge of FIS rules are shown in Table 8. The most common correct responses corresponded to the first (“Self-awareness”), third (“SP inspection”), sixth (“Local safety practices”), seventh (“Take of turns”), and ninth (“Assistance”) questions. Questions related to safe progression (Q2 and Q5) were seldom answered correctly, though the most outstanding lack of knowledge was found for the tenth question (“Identification”).

The influence of age was found to be significant in reference to the correct number of responses to almost every question, but the third and the tenth question had the highest number of correct answers from mainly the senior and adult groups. Similarly, the skill level proved significant in every question but the tenth, with the group of experts showing the highest percentages of correctness for every question. So did the frequency of use of the SP for every question but the fifth and the tenth, with experts reaching the highest levels of knowledge. Neither gender, except for Q4 (males over females), Q7 and Q9 (in both females over males), nor gear used, except for Q3 (skiers over snowboarders) and Q10 (snowboarders over skiers), had a significant influence. 

## 4. Discussion

Past research has shown that accidents in SPs are an important percentage of the total number of accidents taking place in winter resorts and result in relatively more severe injuries than accidents on the slopes [13,14,15]. Therefore, the safety and security of freestylers are an important issue for terrain park management.

It has been found that freestylers’ “personal conditions,” a factor made up of such variables as skill level, attitude—risk-taking, rule observation, etc.—or knowledge of safety rules, is a key factor that has a very important bearing on accident occurrence [10]. In general terms, according to the low levels of active knowledge of existing rules for the SP obtained in this study, it seems advisable for resorts to focus on policies designed to raise the users’ awareness of the fact that the better their personal conditions in terms of knowledge of safety rules for performing freestyle activities, the less likely they are to have an accident; this seems to make it necessary to encourage campaigns informing users about the benefits of knowledge of safety rules [10]. The conduct of SP users highly depends on their level of knowledge regarding existing rules, and a decisive way of reducing accidents would be to ensure that freestylers are sufficiently familiar with the rules contained in the Code of Conduct for Snow Parks issued by the FIS.

To our knowledge, this is the first attempt to specifically assess general perceptions of safety, general acquaintance with existing rules, and actual, active, and passive understanding of the FIS rules for SPs with regard to gender, age, gear used, skill level, and frequency of use of the SP.

The results revealed that 17% and 3% of freestylers feel unsafe—mainly seniors and those who use the SP only occasionally—or very unsafe, respectively, when performing in the SP. Communication of the FIS Rules in the media and at free informative initiatives in snow regions may reduce accidents, consequently improving the feeling of security in SPs. Additionally, eye-catching banners showing the FIS rules should be present at the entrance to the SPs and in adjoining lifts [6].

The present study has also found that young freestylers (aged 12–24) in particular, in contrast to adults and seniors who find them important, tend to disdain rules. In this respect, Ruedl et al. found that risk-taking behavior on ski slopes is associated with youths [25], Carús and Escorihuela found that the highest proportion of the freestyle skiers that sustained injuries in SPs were younger than 20 years of age [12], and Brooks et al. found that the youngest freestyle snowboarders (aged 13–24) were at the highest risk of injury [15]. It suggests that health education should focus on both risk-seeking and safety-seeking behaviors of young freestylers [26]. They could be influenced by including the FIS Code of Conduct for Snow Parks in school lessons, especially if they were required to verify the rules before they were admitted within SPs. This basic knowledge could mean an important policy towards correcting improper behavior.

Sixty three percent of the participants did not know that rules of general application exist in the SPs and, of the freestylers who knew that rules exist, only one-sixth were aware that the rules are called FIS rules. These figures are well under the twenty five percent and the one-third, respectively, previously registered for regular slope users in relation to the ten FIS rules for on slope conduct [6]. Young freestylers, snowboarders, beginners, and those who use the SP only occasionally turned out to be the groups who showed the poorest levels of awareness of the existence of rules. Of the freestylers who knew that rules exist, females, seniors, experts, and those who frequently or always use the SP were the groups who knew best that the existing rules are contained in the FIS Code of Conduct for Snow Parks. In this regard, Hildebrant et al. found that the term FIS Rules is more familiar to experts on slope skiers but, compared to adults, young skiers and seniors were less able to identify it [6]. The newness of the Code could partly account for this remarkable lack of acquaintance with it, however, the stakeholders implied in spreading its knowledge among freestylers (FIS, resorts, ski schools, clubs, etc.) would do well in increasing the quantitative and qualitative suitability of their communication policies for reaching the misinformed groups.

Fortunately, answers to questions devised to assess intuitive behavior of all of the participants for given situations reveal a better knowledge of proper behavior in the SP than what could be inferred from the scarce percentage who could quote rules of the Code for SP’s general application. It suggests that experience and the use of common sense as safety factors while freestyling, amply or outweigh the actual active knowledge of existing rules for SPs. Overall, the appropriate general knowledge of the FIS rules and the intuitive behavior seem to align. More frequently mentioned rules, such as “Look at the terrain,” “Local rules,” “Respect,” or “Assistance” were answered correctly for given situations within the open questions on intuitive behavior, while scarcely mention rules, such as “Train with others,” “Safety equipment,” or “Plan/Understand the progression” were erroneously answered. The lack of knowledge of the latter can be considered especially worrying because they are related to the vast majority of accidents and injuries taking place in SPs, such as those caused by lone freestyler falls and crashes into features or other obstacles as a result of misjudgment of skill, progression, features or safety equipment, and so must be urgently addressed [12,18,27].

It is noticeable that the actual knowledge of rules and the appropriateness of intuitive behavior changes with experience, along age, skill, and frequency of use of the SP. However, young freestylers, who showed the highest level of rejection of rules, the largest gap in active knowledge, and were less able to intuitively implement FIS rules, constitute the group at the highest risk with regard to SP injuries, as previous research has shown [12,15,17,25]. It should be thought advisable that they should attend classes in which instructors ensure students’ knowledge of rules [6]. However, more investigation into SP accidents is needed to highlight hazards, risk-taking behaviors, and causal connections between injuries and disregard of the rules contained in the FIS Code of Conduct for Snow Parks.

Up to now, FIS rules are not enforced legal precepts and only apply in cases of disregard and willful behavior and for insurance purposes [6,22]. However, the aim should be to promote the rules as a measure of prevention of SP accidents [6]. FIS rules comprehend all aspects of safe freestyling and to improve awareness of them, active education campaigns must be undertaken, especially among youths. A variety of stakeholders, such as parents, public institutions, schools, resorts, clubs or rentals, and different strategies should be included. In this sense, it seems that a special educational effort should be made to improve their safety-seeking behaviors, and for that purpose the Canadian Pediatric Society recommends that physicians should provide office-based anticipatory guidance, counselling families to become familiar with and adhere to the Alpine Responsibility Code [28]. Another good example is the US National Ski Areas Association campaign called “Smart Style,” devised to promote education about SP safety among youths [29]. Deeper research should point out the most beneficial policies for the purpose.

The study findings are limited because the ski resort in which it took place did not count on the means to calculate the number of SP users. As well, because some of the counts for 3 × 2 crossings were too small to arrive to meaningful conclusions on independence among groups, within age, skill level and frequency of use of the SP, with respect to actual knowledge of the FIS Rules (e.g., skill level x R1 in Table 7). Another potential limitation of this study is that data have been obtained from freestylers surveyed at a single SP in one country and may not apply to all. Although the results may be considered useful to generate new hypotheses on knowledge of FIS rules for SPs, further research is needed to contrast the characteristics of international participants with different backgrounds. However, this research would furnish stakeholders and particularly ski resort risk managers with valuable information to assess the dimensions of the problems to be avoided or solved through a wide range of possible information and safety policies.

## 5. Conclusions

We found that the great majority of the participants in this study did not know that rules for general application in the SPs exist, that only a small proportion of them were aware that they are called FIS rules, and that, according to answers given to questions devised to assess intuitive behavior, mainly experience and common sense can be trusted as safety factors. The appropriate general knowledge of the FIS rules and the intuitive behavior for given situations seem to align, but the lack of passive knowledge of key rules, such as “Train with others,” “Safety equipment,” or “Plan/Understand the progression” must be addressed at the shortest notice. These findings suggest that effective strategies for the communication of the rules conforming the FIS Code of Conduct for Snow Parks, having younger and more inexperienced freestylers as their main target, must be implemented.

## Figures and Tables

**Table 1:** The rules of the International Ski Federation (FIS) Code of Conduct for Snow Parks.

R1.	Know your own skills and abilities.
R2.	Train with others and work together.
R3.	Look at the terrain before you start.
R4.	Use Safety Equipment Properly.
R5.	Have a Plan.
R6.	Understand the progression of the skill and of the features.
R7.	Observe and follow the local rules.
R8.	Respect the terrain and others.
R9.	Assistance.
R10.	Identification.

**Table 2:** Passive knowledge: intuitive behavior for given situations in the snow parks (SPs).

Q1.	Are you aware of your own skill before choosing jumps, jibs and stunts appropriated to your level?
Q2.	Do you train with others for feedback and faster progression?
Q3.	Do you inspect everything and have an understanding of the conditions of the features and the snow before you start?
Q4.	Do you ensure that you are using safety equipment according to regulations and standards?
Q5.	Do you study skill and features progression and set your goals so that you can move through the progression of both?
Q6.	Do you observe and follow the local rules and safety practices within the SP?
Q7.	Do you wait for features and linking lines to be free before entering them?
Q8.	Can you stand everywhere in the SP?
Q9.	Are you duty bound to assist at accidents?
Q10.	As a responsible party or as a witness, do you exchange personal information following an accident?

Q (1–10): The questions devised to assess passive knowledge.

**Table 3:** Respondents profile.

Profile	N	%
Gender		
Male	296	68
Female	140	32
Age		
Youths (14–24)	134	31
Adults (25–59)	266	61
Seniors (≥60)	36	8
Gear used		
Skis	189	43
Snowboard	247	57
Skill level		
Beginner	17	4
Intermediate	222	51
Expert	197	45
Frequency of use		
Occasionally	54	12
Frequently	252	58
Always	130	30

**Table 4:** Perception of safety among survey participants.

* Safety	Very Safe	Safe	Unsafe	Very Unsafe	*p*
Gender					0.759
Male	97(38)	136(46)	53(18)	10(3)	
Female	42(30)	72(51)	22(16)	4(3)	
Age					<0.001
Youths (14–24)	67(50)	54(40)	8(6)	5(4)	
Adults (25–59)	67(25)	141(53)	51(19)	7(3)	
Seniors (≥60)	5(14)	13(36)	16(44)	2(6)	
Gear used					0.995
Skis	59(31)	91(48)	33(17)	6(3)	
Snowboard	80(32)	117(47)	42(17)	8(3)	
Skill level					0.126
Beginner	5(29)	7(41)	5(29)	0(0)	
Intermediate	65(27)	102(46)	47(21)	8(4)	
Expert	69(35)	99(50)	23(12)	6(3)	
Frequency of use					<0.001
Occasionally	8(15)	19(35)	25(46)	2(4)	
Frequently	67(27)	137(54)	43(17)	5(2)	
Always	64(49)	52(40)	7(5)	7(5)	
Total	139(32)	208(48)	75(17)	14(3)	

* *n* (%).

**Table 5:** Perceived importance of rules among survey participants.

* Importance	Important	Unimportant	*p*
Gender			<0.001
Male	197(67)	99(33)	
Female	116(83)	24(17)	
Age			<0.001
Youths (14–24)	53(40)	81(60)	
Adults (25–59)	225(85)	41(15)	
Seniors (≥60)	35(97)	1(3)	
Gear used			0.565
Skis	133(70)	56(30)	
Snowboard	180(73)	67(27)	
Skill level			<0.001
Beginner	10(59)	7(41)	
Intermediate	143(64)	79(36)	
Expert	160(81)	37(19)	
Frequency of use			0.134
Occasionally	43(80)	11(20)	
Frequently	172(68)	80(32)	
Always	98(75)	32(25)	
Total	313(72)	123(28)	

* *n* (%).

**Table 6:** General knowledge of FIS Rules for SPs among survey participants.

Knowledge	* Rules Exist	*p*	** Named FIS-R	*p*
Gender		0.566		0.251
Male	112(38)		15(13)	
Female	49(35)		11(22)	
Age		0.042		0.003
Youths (14–24)	38(28)		1(3)	
Adults (25–59)	107(40)		20(19)	
Seniors (≥60)	16(44)		5(31)	
Gear used		<0.001		0.265
Skis	90(48)		14(16)	
Snowboard	71(29)		12(17)	
Skill level		<0.001		<0.001
Beginner	1(6)		0(0)	
Intermediate	34(15)		0(0)	
Expert	126(64)		26(21)	
Freq. of use of SP		<0.001		0.123
Occasionally	6(11)		0(0)	
Frequently	92(37)		16(17)	
Always	63(48)		10(16)	
Total	161(37)		26(16)	

* Correct answers: *n* (%). ** Correct answers of those who knew that rules for general application exist: *n* (%).

**Table 7:** Actual active knowledge of existing rules for SPs among survey participants (*).

** FIS Rs	**R1**	***p***	**R2**	***p***	**R3**	***p***	**R4**	***p***	**R5**	***p***
Gender		0.414		0.345		0.719		0.019		0.244
Male	9		3		86		47		12	
Female	14		6		88		27		6	
Age		0.005		.---		0.024		0.128		0.002
Youths (14–24)	8		0		82		37		5	
Adults (25–59)	8		4		87		41		8	
Seniors (≥60)	31		12		94		50		31	
Gear used		0.021		0.184		0.002		0.002		0.974
Skis	13		1		83		44		8	
Snowboard	7		7		90		37		13	
Skill level		.---		.---		<0.001		<0.001		.---
Beginner	0		0		100		100		0	
Intermediate	3		0		13		4		0	
Expert	13		5		87		45		12	
Frequency of use		0.990		.---		<0.001		0.014		0.105
Occasionally	17		0		100		67		0	
Frequently	8		4		83		36		9	
Always	14		3		90		46		13	
Total	11		3		86		41		10	
** FIS Rs	**R6**	***p***	**R7**	***p***	**R8**	***p***	**R9**	***p***	**R10**	***p***
Gender		0.539		0.641		0.239		0.897		0.231
Male	4		58		37		78		6	
Female	6		57		53		86		2	
Age		.---		0.473		<0.001		<0.001		.---
Youths (14–24)	3		66		3		53		0	
Adults (25–59)	4		54		49		88		3	
Seniors (≥60)	12		62		94		94		31	
Gear used		0.131		0.022		0.899		0.198		0.076
Skis	6		56		32		69		1	
Snowboard	3		61		55		94		10	
Skill level		.---		<0.001		<0.001		<0.001		.---
Beginner	0		0		0		0		0	
Intermediate	0		12		6		10		0	
Expert	5		52		44		85		6	
Frequency of use		.---		0.003		0.195		<0.001		.---
Occasionally	17		33		67		67		0	
Frequently	2		65		45		78		3	
Always	6		49		37		84		9	
Total	4		58		42		80		5	

(*) Cited FIS Rules (data in %). ** R (1–10): Rules of the FIS Code of Conduct for Snow Parks.

**Table 8:** Actual passive knowledge of existing rules for SPs among survey participants (*).

** Questions	**Q1**	***p***	**Q2**	***p***	**Q3**	***p***	**Q4**	***p***	**Q5**	***p***
Gender		0.959		0.928		0.190		0.002		0.997
Male	67		8		58		35		6	
Female	67		8		51		20		6	
Age		<0.001		0.003		0.848		<0.001		0.004
Youths (14–24)	46		3		57		18		1	
Adults (25–59)	75		9		55		34		9	
Seniors (≥60)	86		19		58		47		6	
Gear used		0.060		0.677		0.002		0.223		0.957
Skis	74		7		65		27		6	
Snowboard	62		8		49		32		6	
Skill level		<0.001		<0.001		<0.001		<0.001		<0.001
Beginner	0		0		18		6		0	
Intermediate	49		2		42		7		2	
Expert	93		16		75		58		12	
Frequency of use		<0.001		<0.001		<0.001		<0.001		0.067
Occasionally	46		2		26		15		0	
Frequently	64		5		59		22		6	
Always	81		16		63		52		9	
Total	67		8		56		30		6	
** Questions	**Q6**	***p***	**Q7**	***p***	**Q8**	***p***	**Q9**	***p***	**Q10**	***p***
Gender		0.191		<0.001		0.557		<0.001		0.423
Male	66		44		39		70		2	
Female	72		72		42		95		3	
Age		<0.001		<0.001		<0.001		<0.001		.---
Youths (14–24)	55		31		19		55		0	
Adults (25–59)	71		62		47		86		2	
Seniors (≥60)	94		72		72		100		11	
Gear used		0.372		0.167		0.176		0.578		0.049
Skis	66		49		37		77		1	
Snowboard	70		56		43		79		3	
Skill level		<0.001		<0.001		<0.001		0.001		.---
Beginner	0		6		12		47		0	
Intermediate	49		34		24		75		0	
Expert	95		79		61		84		5	
Frequency of use		<0.001		<0.001		<0.001		0.004		0.595
Occasionally	41		37		26		69		4	
Frequently	66		47		36		75		2	
Always	83		72		55		88		2	
Total	68		58		40		78		2	

(*) Correct answers (data in %). ** Q (1–10): Questions devised to assess passive knowledge.
